# Discrepant comorbidity between minority and white suicides: a national multiple cause-of-death analysis

**DOI:** 10.1186/1471-244X-9-10

**Published:** 2009-03-18

**Authors:** Ian RH Rockett, Yinjuan Lian, Steven Stack, Alan M Ducatman, Shuhui Wang

**Affiliations:** 1 Injury Control Research Center, West Virginia University, Morgantown, West Virginia, USA; 2 Department of Community Medicine, West Virginia University, West Virginia, USA; 3 Epidemiology Data Center, University of Pittsburgh, Pittsburgh, Pennsylvania, USA; 4 Departments of Criminal Justice and Neuropsychiatry, Wayne State University, Detroit, Michigan, USA; 5 Health Care Informatics, State of Tennessee, Bureau of TennCare, Nashville, Tennessee, USA

## Abstract

**Background:**

Clinician training deficits and a low and declining autopsy rate adversely impact the quality of death certificates in the United States. Self-report and records data for the general population indicate that proximate mental and physical health of minority suicides was at least as poor as that of white suicides.

**Methods:**

This cross-sectional mortality study uses data from Multiple Cause-of-Death (MCOD) public use files for 1999–2003 to describe and evaluate comorbidity among black, Hispanic, and white suicides. Unintentional injury decedents are the referent for multivariate analyses.

**Results:**

One or more mentions of comorbid psychopathology are documented on the death certificates of 8% of white male suicides compared to 4% and 3% of black and Hispanic counterparts, respectively. Corresponding female figures are 10%, 8%, and 6%. Racial-ethnic discrepancies in the prevalence of comorbid physical disease are more attenuated. Cross-validation with National Violent Death Reporting System data reveals high relative underenumeration of comorbid depression/mood disorders and high relative overenumeration of schizophrenia on the death certificates of both minorities. In all three racial-ethnic groups, suicide is positively associated with depression/mood disorders [whites: adjusted odds ratio (AOR) = 31.9, 95% CI = 29.80–34.13; blacks: AOR = 60.9, 95% CI = 42.80–86.63; Hispanics: AOR = 34.7, 95% CI = 23.36–51.62] and schizophrenia [whites: AOR = 2.4, 95% CI = 2.07–2.86; blacks: AOR = 4.2, 95% CI = 2.73–6.37; Hispanics: AOR = 4.1, 95% CI = 2.01–8.22]. Suicide is positively associated with cancer in whites [AOR = 1.8, 95% CI = 1.69–1.93] and blacks [AOR = 1.8, 95% CI = 1.36–2.48], but not with HIV or alcohol and other substance use disorders in any group under review.

**Conclusion:**

The multivariate analyses indicate high consistency in predicting suicide-associated comorbidities across racial-ethnic groups using MCOD data. However, low prevalence of documented comorbid psychopathology in suicides, and concomitant racial-ethnic discrepancies underscore the need for training in death certification, and routinization and standardization of timely psychological autopsies in all cases of suicide, suspected suicide, and other traumatic deaths of equivocal cause.

## Background

Suicide kills far more Americans than homicide, its twin and much higher profile cause of fatal intentional injury. For 1999–2003, the observation period for this study, the annualized suicide rate for the United States was almost double the homicide rate – 11 versus 6 per 100,000 population [1]. Suicide ranked fourth as a cause of potential years of life lost before age 65 years for (non-Hispanic) whites, seventh for Hispanics, and ninth for (non-Hispanic) blacks. Suicide is avoidable mortality [2,3], and the proximate mental and physical health of decedents is salient to its understanding. To better comprehend the relationship between proximate health and suicide, we accessed national vital statistics data to describe and evaluate comorbidity in white, black, and Hispanic suicides. Untenable as biological constructs, race and ethnicity are social constructs with important implications for health disparities and healthcare delivery [4]. Questions about the reliability and validity of multiple cause-of-death data added impetus to our research.

Although we assume that black and Hispanic suicides experienced at least as much proximate mental and physical illness as white suicides, we expect that medicolegal authorities record less comorbidity on their death certificates. This assumption and expectation stem primarily from comparative survey research on the general US population. A study utilizing the Sample Adult component of the 1998–2003 National Health Interview Surveys showed that blacks and Hispanics report being in worse health, having more physical limitations and annual bed days, less health insurance coverage, fewer physician visits, and more unmet mental healthcare needs than whites [5]. These groups also compare unfavorably on objective measures of healthcare access and management [6]. With the focus more specifically on mental health, an analysis of the 2005–2006 National and Nutrition Examination Survey showed that blacks had a higher prevalence of depression than whites and Hispanics [7]. An analysis of the 2001–2004 National Health Interview Survey indicated no difference in the prevalence of serious psychological distress between blacks and whites [8]. While the prevalence was equivalent between Hispanics and whites at ages 18 through 64 years, it was almost three times higher for Hispanics at ages 65 and older. Limited to a comparison between blacks and whites, the National Survey of American Life revealed 45% excess chronicity in major depressive disorder among blacks [9]. Turning back to racial-ethnic treatment disparities, an analysis of the Collaborative Psychiatric Epidemiology Surveys found that 36% of Latinos and 41% of African-Americans with a past-year depressive disorder had received mental health treatment compared to 60% of non-Latino whites [10].

Our expectation of relative underdocumentation of comorbidity on the death certificates of the minority suicides is partially supported by a records-based study [11]. Subjects were adult enrollees in TennCare, Tennessee's Medicaid-waiver managed care program, a public assistance program which offers equal financial access to all low-income groups [12]. This study found that only 29% of African-American suicides and possible suicides, as compared to 51% of white opposites, used prescribed antidepressants in the year prior to their deaths [11]. The authors interpreted this differential as indirect evidence that serious mood disorders are underdiagnosed or undertreated in African-Americans. However, they acknowledged that African-Americans may have been more likely to receive nonpharmacological therapy than whites, and thus could have been less likely to have antidepressant prescriptions filled. They operationalized their possible suicides as decedents whose deaths were officially classified under injury of undetermined intent, and no subjects had a record of chronic psychosis or serious medical illness.

Meta-analyses which combine studies of predominantly patient samples affirm that both comorbid psychopathology and physical disease are suicide determinants [13-16]. Reinforcement comes from two national multiple cause-of-death (MCOD) analyses, an Australian suicide study [17] and a US replication [18]. Implying high bi-national reliability of MCOD data, comparison of their multivariate results revealed identical suicide-associated comorbidities: depression and mood disorders, schizophrenia, and cancer. By contrast, gender-specific prevalences of comorbid psychopathology were almost three times as high in Australian suicides. Moreover, the prevalence of comorbid physical disease was twice as high in Australian as US male suicides, and there was 78% excess prevalence among Australian female suicides compared to US counterparts. The magnitude of these prevalence gaps was surprising, as was the revelation that only among Australian suicides was comorbid psychopathology more prevalent than comorbid physical disease. While both nations are economically and technologically sophisticated democracies, with a shared suicide rate of 11 per 100,000 population [1,19], life expectancy and healthy life expectancy at birth in Australia exceed US expectancies by approximately three years [20,21]. Indeed, the prevalence gaps and differential weight of comorbid psychopathology indicate that Australian death certificates surpass those of the United States in documenting true comorbidity among suicides. We subsequently ascertained that Australian death investigations are more standardized than those in the United States, and that US coders have less capacity than Australian counterparts to access medicolegal records to help resolve outstanding questions [[22]; personal communication, Robert N. Anderson, National Center for Health Statistics, September 22, 2008].

Death certificates are essential for conducting epidemiologic surveillance, planning and prioritizing healthcare expenditures and services, and formulating policy for preventing traumatic deaths at state and national levels. Moreover, mortality data from the National Vital Statistics System are more universal, standardized, and timely than data from other major health databases [23]. Nevertheless, critics cite persuasive empirical evidence from validation studies to charge that death certificates are seriously deficient [24-27], In the mid-1990s, a survey of over 700 medical examiners and coroners showed that few had received any formal training in death certification in their medical school training or residencies [28]. Death certification still receives scant attention in medical school and hospital training [29-31]. An additional complicating factor is the declining adult autopsy rate, from 18% in 1955 to just 8% by 2003 [32]. Both a low autopsy rate and training deficits will impede ascertainment and recording of comorbidity on the death certificates of suicides. We are motivated by these factors and the US-Australian prevalence gaps, as well as by the healthcare utilization literature, to assess whether comorbidity is underdocumented in black and Hispanic suicides relative to white suicides.

## Methods

MCOD public use files from the National Vital Statistics System within the National Center for Health Statistics are our data source for this cross-sectional mortality study. To help stabilize age-, gender-, and racial-ethnic-specific comorbidity prevalence estimates, we chose a five-year observation period, 1999–2003. Causes of death were precoded under the *International Statistical Classification of Diseases and Related Health Problems, Tenth Revision ICD-10 *[33]. Our study group comprised white, black, and Hispanic decedents whose underlying cause of death was intentional self-harm (X60–X84) or sequelae of intentional self-harm (Y87.0).

We employed a comparison group in order to identify suicide-associated comorbidities through multivariate analysis. This group comprised white, black, and Hispanic decedents whose underlying cause of death was unintentional injury (V01–X59), sequelae of transport unintentional injury (Y85), or sequelae of other unintentional injury (Y86). We further confined our study and comparison groups to persons who died at ages 15 years or older in the 50 states and the District of Columbia. Fifteen years was the minimum age cutoff because suicide was rarely reported among children and younger teens [34], who constituted less than 1% of all suicides during the observation period. [1] Relevant given the nature of the racial-ethnic comparisons in this study, Hispanic blacks comprised only 1% of Hispanic suicide subjects. This finding holds independent of gender.

Suicide almost invariably becomes the official underlying cause of death when medicolegal authorities rule a death as suicide in a timely manner [35]. Concordance between a suicidal mention on the death certificate and its assignment as the underlying cause approaches 100% [36]. Unlike deaths attributed to a chronic disease, comorbidity enumerated on the death certificates of suicides is almost necessarily antecedent owing to the typically acute nature of injury mortality. However, antecedence does not imply causality.

Replicating the methods of the prior US MCOD suicide study [18] in a racial-ethnic context, we disaggregated comorbidity into 14 types within two major subheadings: mental/behavioral disorders and physical disease. Our prevalence calculations drew upon all potential comorbid mentions on a death certificate – up to 20. However, we counted only one condition where more than one was associated with a particular type of comorbidity. Each of the 14 types served as a distinct outcome variable in a series of unconditional logistic regression analyses that we performed to identify suicide-associated comorbidities in the MCOD dataset for whites, blacks, and Hispanics, respectively. We adjusted the odds ratios for age (three ordinal categories: 15–34, 35–64, and 65+ years) and gender. Our results provide a preliminary basis for assessing the reliability of the comorbidity data in relation to suicide. In a sensitivity analysis we eliminated our covariates, age and gender, from the model. All variables derived from the MCOD database.

Sampling variability was not an issue in this study since the data represented complete counts. However, to allow for random variation in our cross-tabulated prevalence data on suicides, we conducted two series of Chi-square tests before reporting on differentials and patterns. Our first series were two-by-three tests. For each category of comorbidity, distinguishing presence or absence, we separately assessed statistically significant variation within gender and across racial-ethnic groups and age groups, respectively. These results justified our series of two-by-two tests. In these tests we compared white suicides with black suicides and Hispanic suicides, respectively, within specific comorbidity categories for all ages and also within each of our three age groups for each gender. Then separately for each racial-ethnic group within gender, we similarly compared cause-specific comorbidity prevalences across these age groups. To assess change in prevalence across age, we simply compared the youngest age group with the middle age group and then the middle group with the oldest group. In the prevalence tables, we identified each estimate which was calculated on the basis of a cell size with fewer than 20 cases.

To address the question of whether comorbidity was relatively underdocumented on the death certificates of minority suicides, we cross-validated selected prevalence data from our study with corresponding data for 2004 from a CDC (Centers for Disease Control and Prevention) study which utilized the US National Violent Death Reporting System (NVDRS) [37]. Approximating a real-time environment, the NVDRS combines data from the offices of coroners and medical examiners, police reports, death certificates, toxicology laboratories, and interviews with family and friends of the decedents. Our cross-validation was confined to schizophrenia and depression and mood disorders, given accessible NVDRS data. We computed black-to-white and Hispanic-to-white prevalence ratios for a given type of comorbidity in each study. Then for the two minorities separately, we calculated the ratio difference as a percentage of the initial ratio, which served as the criterion standard. The product estimated the degree of underreporting or overreporting of a condition among black or Hispanic suicides relative to white suicides. Statistical analyses were conducted using SAS (version 9.13, Cary, NC).

## Results

### Prevalence of comorbidity

We first report on differentials in the prevalence of comorbidity documented on death certificates between white and minority suicides and also on within-group patterns (Tables 1, 2, 3). We highlight only statistically significant variations ($\chi_{1}^{2}$; p < 0.05). Psychopathology is more than twice as prevalent among white male suicides compared to minority counterparts (Table 1). Specifically, 8% of white male suicides have at least one mention of comorbid psychopathology documented on their death certificate, compared to 4% of black male suicides and 3% of Hispanic male suicides. Corresponding figures for female suicides are 10%, 8%, and 6%. While manifesting less minority-majority divergence than in the case of comorbid psychopathology, the prevalence of comorbid physical disease documented on the death certificates of male suicides is lower for blacks and Hispanics than for whites. Among female suicides, the prevalence is only lower than whites for Hispanics. Confined to subjects with comorbidity documented on their death certificates, mean number of mentions does not vary with majority-minority status.

**Table 1:** Comorbid medical conditions in suicides by gender, racial-ethnicity, category, and measure, United States, 1999–2003

**Medical Condition (ICD-10)/Measure**	**Males**			**Females**		
		
	**White**	**Black**	**Hispanic**	**White**	**Black**	**Hispanic**
**Number of Subjects**	**101,804**	**7,827**	**7,825**	**25,478**	**1,563**	**1,349**
**Mental/Behavioral Disorders (F00–F99)**						
mean number of mentions*	1.2	1.2	1.2	1.2	1.3	1.2
% of all mentions	43.9%	35.4%	30.0%	42.8%	39.0%	39.6%
% of total suicides**	7.8%	3.8%	3.1%	10.5%	7.7%	6.4%
**Physical Diseases (A00–E90; G00–R99)**						
mean number of mentions*	1.7	1.4	1.5	1.8	1.8	1.6
% of all mentions	56.1%	64.6%	70.0%	57.2%	61.0%	60.4%
% of total suicides**	7.3%	5.7%	5.5%	9.5%	8.5%	7.4%
**Total Disorders/Diseases**						
mean number of mentions*	1.4	1.4	1.4	1.5	1.6	1.4
% of suicides with 2+ mentions	6.7%	3.3%	3.2%	9.7%	9.5%	5.5%

* Calculated only for subjects with at least one mention of a comorbid condition on their death certificates.

**Percentage of suicides with at least one mention of that comorbidity category on their death certificates.

**Table 2:** Prevalence of comorbid medical conditions in male suicides by age, racial-ethnicity, category, and type: United States, 1999–2003

	**Whites**				**Blacks**				Hispanics*			
			
**Medical Condition (ICD-10)**	**Prevalence per 100**				**Prevalence per 100**				**Prevalence per 100**			
	
**Age (in years)**	**15–34**	**35–64**	**65+**	**Total**	**15–34**	**35–64**	**65+**	**Total**	**15–34**	**35–64**	**65+**	**Total**
**Number of deaths**	**27931**	**52814**	**21059**	**101804**	**4029**	**3174**	**624**	**7827**	**3927**	**3194**	**704**	**7825**
**Mental/Behavioral Disorders**	9.0	10.6	6.9	9.4	3.8	5.4	4.2	4.5	3.3	4.0	2.3**	3.5
Organic (F00–F09)	0.1	0.1	0.2	0.1	0.1**	0.0**	0.3	0.1	0.0**	0.1**	0.0**	0.1**
Alcohol use (F10)	2.5	3.3	0.7	2.6	1.2	1.4	0.5	1.2	1.2	1.7	0.3**	1.3
Other substance use (F11–F19)	1.0	0.8	0.3	0.7	0.5	1.0	0.2	0.7	0.7	0.4**	0.0**	0.5
Schizophrenia (F20–F29)	0.4	0.3	0.1	0.3	0.5	0.6	0.0	0.5	0.2**	0.2**	0.0**	0.2**
Mood disorder/depression (F30–F39)	4.7	5.7	5.5	5.4	1.4	2.3	3.2	1.9	1.1	1.3	2.0**	1.3
Other mental health (F40–F99)	0.4	0.4	0.2	0.3	0.1**	0.1**	0.0**	0.1**	0.0**	0.2**	0.0**	0.1**
**Physical Diseases**	6.3	9.7	19.5	10.8	5.2	8.5	16.8	7.5	6.3	7.4	14.5	7.5
HIV (B20–B24)	0.0**	0.1	0.0**	0.1	0.1**	0.2**	0.0	0.1**	0.1**	0.2**	0.1**	0.1**
Other communicable(A+B residual)	0.1	0.2	0.2	0.2	0.1**	0.4**	0.5	0.3	0.2**	0.1**	0.1**	0.2**
Cancer (C00–C99)	0.0**	0.7	4.9	1.4	0.0**	0.6**	5.1	0.7	0.0**	0.1**	2.7**	0.3
Nervous system (G00–G99)	2.0	1.5	1.7	1.7	1.5	1.2	0.3	1.3	2.4	1.8	1.4**	2.1
Circulatory (I00–I99)	1.3	2.8	6.2	3.1	1.3	2.3	4.5	2.0	0.8	1.8	5.4	1.6
Respiratory (J00–J99)	0.5	0.9	2.8	1.3	0.4	1.0	2.1	0.9	0.6	0.6**	1.6**	0.7
Digestive (K00–K99)	0.3	0.7	0.5	0.6	0.1**	0.3	0.5**	0.3	0.4**	0.9	0.1**	0.7
Other diseases (D,E,H,L-R)	1.9	2.7	3.3	2.9	1.6	2.6	3.8	2.4	1.8	2.0	3.0	2.1
**Total Disorders and Diseases**	**15.3**	**20.2**	**26.4**	**20.2**	**9.0**	**13.9**	**21.0**	**11.9**	**9.5**	**11.4**	**16.8**	**10.9**

* Percentage black is 1.2.

**Prevalence estimate may be unreliable since it is based on a cell size with less than 20 cases.

**Table 3:** Prevalence of comorbid medical conditions in female suicides by age, racial-ethnicity, category, and type: United States, 1999–2003

	**Whites**				**Blacks**				Hispanics*			
			
**Medical Condition (ICD-10)**	**Prevalence per 100**				**Prevalence per 100**				**Prevalence per 100**			
	
**Age (in years)**	**15–34**	**35–64**	**65+**	**Total**	**15–34**	**35–64**	**65+**	**Total**	**15–34**	**35–64**	**65+**	**Total**
**Number of deaths**	**5598**	**16087**	**3793**	**25478**	**602**	**835**	**126**	**1563**	**627**	**630**	**92**	**1349**
**Mental/Behavioral Disorders**	11.3	13.4	9.9	12.4	7.0	11.4	11.1**	9.7	4.6	9.8	7.6**	7.3
Organic (F00–F09)	0.1**	0.1**	0.3**	0.1	0.0**	0.2**	1.6**	0.3**	0.0**	0.0**	0.0**	0.0**
Alcohol use (F10)	2.1	3.5	1.1	2.8	2.0**	2.5	0.8**	2.2	0.8**	3.7	1.1**	2.1
Other substance use (F11–F19)	1.4	1.2	0.8	1.2	1.8**	1.6**	0.0**	1.5	0.3**	1.0**	2.2**	0.7**
Schizophrenia (F20–F29)	0.4	0.4	0.2**	0.3	0.2**	1.0**	0.8**	0.6**	0.2**	0.3**	0.0**	0.2**
Mood disorder/depression (F30–F39)	6.7	7.8	7.1	7.4	2.8**	5.7	7.9**	4.8	2.7**	4.6	4.3**	3.7
Other mental health (F40–F99)	0.6	0.5	0.4**	0.5	0.2**	0.4**	0.1**	0.3**	0.6**	0.3**	0.0**	0.4**
**Physical Diseases**	9.6	14.9	21.9	14.8	8.8	14.4	27.0	13.2	10.0	10.5	13.0**	10.5
HIV (B20–B24)	0.0**	0.0**	0.0**	0.0**	0.0**	0.4**	0.0**	0.2**	0.0**	0.0**	0.0**	0.0**
Other communicable (A+B residual)	0.2**	0.5	0.4**	0.4	0.0**	0.6**	0.0**	0.3**	0.2**	0.2**	0.0**	0.1**
Cancer (C00–C99)	0.1**	0.7	2.5	0.8	0.2**	0.5**	1.6**	0.4**	0.0**	0.5**	0.0**	0.2**
Nervous system (G00–G99)	2.5	2.1	2.0	2.1	1.7**	1.2**	2.4**	1.5	3.7	2.2**	1.1**	2.8
Circulatory (I00–I99)	1.6	4.0	7.5	4.0	1.7**	5.1	11.1**	4.3	1.1**	2.5**	5.4**	2.1
Respiratory (J00–J99)	1.1	1.8	3.6	2.2	1.2**	1.6**	2.4**	1.5	0.6**	1.1**	2.2**	1.1**
Digestive (K00–K99)	1.3	1.4	0.9	1.4	1.7**	1.8**	0.8**	1.9	2.1**	1.7**	1.1**	2.0
Other diseases (D,E,H,L-R)	2.9	4.5	5.1	5.0	2.5**	3.2	8.7**	4.4	2.4**	2.2**	3.3**	2.9
**Total Disorders and Diseases**	**20.9**	**28.3**	**31.8**	**27.2**	**15.8**	**25.7**	**38.1**	**22.9**	**14.7**	**20.3**	**20.7**	**17.7**

* Percentage black is 1.3.

**Prevalence estimate may be unreliable since it is based on a cell size with less than 20 cases.

Tables 2 (males) and 3 (females) show the prevalence of documented comorbid psychopathology and physical disease disaggregated by age and racial-ethnicity. Comorbidity as a whole increases monotonically with age except among Hispanic female suicides. Comorbid physical disease peaks at the oldest ages. By contrast, comorbid psychopathology peaks at middle age except among black female suicides. Hispanic males aside, mood disorders and depression predominate among psychopathologies irrespective of gender. These conditions are four times more prevalent among white male suicides than corresponding Hispanics and nearly three times more prevalent than in corresponding blacks. The prevalence of mood disorders and depression in white female suicides is twice as high as in Hispanic counterparts and 1.5 times higher than in black counterparts. The prevalence of circulatory disease is relatively high among black female suicides and white suicides of both genders.

### Suicide-associated comorbidities

With odds ratios adjusted for age and sex, and unintentional injury decedents as the referent, logistic regression analyses of suicide-associated comorbidities reveal many similarities and some differences across racial-ethnic groups (Table 4). Suicide is positively associated with schizophrenia and mood disorders and depression in all three racial-ethnic groups. It is positively associated with cancer among whites and blacks only, and with residual mental health disorders among whites and Hispanics. A positive link between suicide and nervous system disease is limited to Hispanics. Although the point-estimated odds ratio for suicide-associated mood disorders and depression is strikingly high across all three racial-ethnic groups, black suicides are 61 times more likely than black unintentional injury decedents to have such diagnoses recorded on their death certificates. Main results are not modified by the elimination of age and gender as covariates from the basic multivariate model.

**Table 4:** Logistic regression results for suicide-associated comorbidities in three racial-ethnic groups: United States, 1999–2003

	**Whites**			**Blacks**			**Hispanics**		
			
**Medical Condition (ICD-10)**	**Odds** Ratio*	**95% CI (Wald)**		**Odds** **Ratio**	**95% CI (Wald)**		**Odds** Ratio*	**95% CI (Wald)**	
**Mental/Behavioral Disorders**									
Organic (F00–F09)	0.1	0.07	0.11	0.3	0.13	0.54	0.2	0.07	0.49
Alcohol Use (F10)	0.5	0.49	0.53	0.3	0.26	0.38	0.3	0.25	0.37
Other substance use (F11–F19)	0.2	0.18	0.21	0.1	0.09	0.15	0.1	0.09	0.16
Schizophrenia (F20–F29)	2.4 **	2.07	2.86	4.2 **	2.73	6.37	4.1 **	2.01	8.22
Mood disorder/depression (F30–F39)	31.9**	29.80	34.13	60.9 **	42.80	86.63	34.7 **	23.36	51.62
Other mental health (F40–F99)	1.7 **	1.43	1.94	1.1	0.55	2.31	2.6 **	1.24	5.45
**Physical Disease**									
HIV (B20–B24)	1.0	0.76	1.45	0.5	0.29	1.02	1.3	0.54	2.98
Other communicable (A+B residual)	0.2	0.17	0.23	0.3	0.19	0.43	0.2	0.15	0.42
Cancer (C00–C99)	1.8 **	1.69	1.93	1.8 **	1.36	2.48	1.2	0.74	1.78
Nervous system (G00–G99)	0.7	0.65	0.72	0.9	0.71	1.05	1.5 **	1.24	1.72
Circulatory (I00–I99)	0.2	0.20	0.22	0.2	0.20	0.25	0.2	0.20	0.27
Respiratory (J00–J99)	0.2	0.18	0.20	0.2	0.20	0.31	0.3	0.22	0.37
Digestive (K00–K99)	0.8	0.61	0.71	0.5	0.39	0.74	0.6	0.42	0.71
Other diseases (D,E,H,L-R)	0.3	0.30	0.33	0.4	0.31	0.42	0.4	0.36	0.50

* Odds ratios adjusted for gender and categorical age (15–34; 35–64; 65+ years). Unintentional injury decedents served as the referent in all 14 logistic regression analyses for each racial-ethnic group.

**Suicide is positively associated with that type of comorbidity.

### Cross-validation

In comparing our prevalence data with the criterion standard, NVDRS data, we are reporting on the two psychopathologies positively associated with suicide in each of the three study groups; namely, mood disorders and depression, and schizophrenia. White suicides in the NVDRS register seven times more comorbid mood disorders and depression, under an additivity assumption, and 14 times more comorbid schizophrenia than white counterparts in the MCOD dataset (Table 5). Respective black-to-white and Hispanic-to-white prevalence ratios indicate substantial underreporting in the MCOD dataset of comorbid mood disorders and depression for minority suicides relative to white suicides. By contrast, there is evidence of high relative overreporting of comorbid schizophrenia.

**Table 5:** Prevalence, prevalence ratios, and relative misreporting of selected comorbid mental disorders in black and Hispanic suicides using two data sources, United States

	National Violent Death Reporting System*			Multiple Cause-of-Death Data**		
**Mental Disorder**	**Prevalence**	**Prevalence Ratio**		**Prevalence**	**Prevalence Ratio**	
	**White**	**Black/White**	**Hispanic/White**	**White**	**Black/White**	**Hispanic/White**
	**(1)**	**(2)**	**(3)**	**(4)**	**(5)**	**(6)**
Mood disorder/depression	34.7%	0.6	0.5	4.7%	0.4	0.3
Schizophrenia	4.2%	0.6	0.3	0.3%	1.7	0.7

	**Relative Misreporting of Prevalence in Multiple Cause-of-Death Data**					

	**Blacks**			**Hispanics**		
	**({5} - {2} ÷ {2}) × 100**			**({6} - {3} ÷ {3}) × 100**		
Mood disorder/depression	-33%	-40%				
Schizophrenia	183%	133%				

* Data for 2004.

**Data for 1999–2003.

## Discussion

Not limited to patient populations, MCOD data offer a unique and enduring record of comorbidity among completed suicides in the United States. We used these data for the period 1999–2003 to compare and critically evaluate comorbidity among suicides in three racial-ethnic groups: whites, blacks, and Hispanics. As we expected, but contrary to health disparities in the general population, less comorbid psychopathology is documented on the death certificates of black and Hispanic suicides than those of white counterparts. Collectively, our study and its predecessors [17,18] make us rather sanguine about the reliability of MCOD data in predicting suicide-associated comorbidities. By contrast, we are skeptical about the validity or, more particularly, the sensitivity of comorbidity that is documented on the death certificates of suicides. Resting on limited cross-validation with NVDRS data [37], we infer that mood disorders and depression, although not schizophrenia, are profoundly underdocumented on death certificates for blacks and Hispanics relative to whites.

Even the NVDRS is likely to be wanting in the documentation of comorbid psychopathology among suicides. For if we emphasize the absolute instead of the relative, psychological autopsy studies provide compelling evidence that comorbid psychopathology is probably grossly under-documented on the death certificates of suicides irrespective of racial-ethnicity and type of disorder. A meta-analysis of 27 of these in-depth studies of record reviews and interviews with survivors estimated that 87% of suicides had a mental disorder [38]. A systematic review, which included meta-analyses conducted separately on 22 case-control studies and 54 case series, reported 90% prevalence of mental disorders in suicide cases compared to 27% in controls, and 91% prevalence in suicides from the case series [39]. Few of the case-control studies involved US adults.

HIV was not a determinant of suicide in any of the three racial-ethnic groups in our study, even though a 1994 meta-analysis identified this disease as a risk factor [13]. This finding more refinedly affirmed such a finding from the first US MCOD suicide study [18]. Posited by the authors of that study as an ex post facto explanation, receipt of effective antiviral therapy could have altered the mindset of HIV-positives by removing a hitherto imminent and grave threat to survival. Thus, if tenable, minority-majority status may not modify this explanation. To that point, another recent US study attributed a relative reduction in HIV mortality to some closure of the life expectancy gap separating blacks from whites [40]. Nevertheless, blacks and Hispanics continue to bear a disproportionate share of the diminishing national HIV/AIDS burden [41,42]. The absence of HIV as a suicide determinant in these groups may be a marker of success by the healthcare system in delivering appropriate therapy to minority HIV-positives. Epidemiologists and social scientists may further clarify the relationship between HIV and suicide utilizing longitudinal data.

A longitudinal evaluation of race and Hispanic origin on death certificates indicated excellent agreement between self-report and death certificate proxy report for blacks and whites and good agreement for Hispanic subgroups [43]. Nevertheless, our results could have been distorted by racial-ethnic heterogeneity, such as between African-Americans and black Caribbean Americans and native-born and foreign-born Hispanics, respectively [5,9,44,45]. For example, an analysis of the 2001–2002 National Epidemiologic Survey on Alcohol and Related Conditions showed that Mexican-Americans manifested a lower lifetime risk of psychiatric comorbidity than US-born non-Hispanic whites [46]. There was, however, no differentiation of risk among their foreign-born counterparts.

Racial-ethnic variation in the respective prevalences of comorbid psychopathology and physical disease may be real, and simply reflect differences in suicide etiology [47-53]. Alternatively, the data are consistent with our expectation that this variation may be partially or wholly artifactual. Such artifactuality would likely be a function of interactions between family, friends, and acquaintances of decedents with medicolegal authorities, variations in social pressure to withhold psychological information across racial and ethnic groups, and differential fastidiousness with which authorities compile medical records on suicides. However, this research could not assess the merits or relative contributions of these factors. Beyond the highly restricted comparisons, our cross-validation of the documentation of comorbid psychopathology on death certificates of suicides was further constrained by the fact that the NVDRS covers only a minority of states – 14 states in 2004 and 17 currently [54]. Moreover, this system comingles personal reports from family and friends on the proximate mental status of decedents with diagnostic data. Thus, any improvement in the sensitivity of documentation of comorbid psychopathology among suicides, rendered by the NVDRS, likely means diminished specificity.

Gaps between minority and white suicides in comorbidity prevalence, which we estimated from MCOD data, are attenuated for physical disease compared to psychopathology. While unable to validate a relative data deficit for any physical disease, as we did for two types of psychopathology, the observed gaps are congruent with previously documented and variable healthcare utilization and access. Returning to psychopathology, we found no association between alcohol use or other substance use disorders and suicide – a result mirroring findings from prior MCOD studies [17,18]. These relationships are probably complex. For example, an analysis of data from the 1993 US National Mortality Followback Survey showed that problem drinking was positively associated with suicide in whites but negatively associated in blacks [55]. A cross-national systematic review of the relationship between alcohol use disorders and injury in hospital emergency department patients inferred a weak association [56]. In our study, positive links between both alcohol and other substance use disorders and suicide may have been masked by the universal strength of the depression-suicide nexus or by our conventional choice of unintentional injury decedents as the control population. A study limitation is that a change in the coding of education in the MCOD dataset for 2003 precluded us from incorporating educational attainment as a covariate, and as the only potentially suitable proxy for socioeconomic status, in our regression analyses.

## Conclusion

A comprehensive and accurate record of comorbidity on death certificates would advance suicide surveillance well beyond the capacity afforded by underlying cause-of-death information alone. Researchers could utilize enhanced data to fortify the evidence base for suicide, not only through improving description, but by better informing etiology, policy, prevention, and evaluation. Substantial amelioration of likely relevant comorbidity documentation for all racial-ethnic groups would follow from a formal commitment by medical schools and teaching hospitals to train students and residents in death certification, implementation of equivalent in-service training for practicing providers, and routinization and standardization of timely psychological autopsies in all cases of suicide, suspected suicide, and other traumatic deaths of equivocal cause. While these actions would improve understanding of suicide and lead to more effective interventions, we predict that some racial-ethnic gaps in the documentation of comorbidity among suicides will persist as long as access to and utilization of healthcare remain incommensurate with need. We plan to expand our research by examining comorbidity among possible suicides. Results may have implications for improving the sensitivity of suicide certification across racial-ethnic groups. Attention has been drawn to a racial paradox; namely, that while blacks manifest a risk for suicide equal to or greater than that of whites, their suicide rate is less than half [57]. Variable ascertainment of comorbid depression or cancer, for example, might contribute to this paradox through differentially affecting the tipping point where authorities rule a true suicide a suicide.

## Competing interests

The authors declare that they have no competing interests.

## Authors' contributions

IRHR and SS conceived and designed the study. IRHR obtained the data and the funding. IRHR, YL, and SW managed the data. All authors analyzed and interpreted the data, participated in drafting the MS, and read and approved the final version.

## Pre-publication history

The pre-publication history for this paper can be accessed here:


